# Water Extraction Kinetics of Bioactive Compounds of *Fucus vesiculosus*

**DOI:** 10.3390/molecules24183408

**Published:** 2019-09-19

**Authors:** Ricardo M. Ferreira, Ana Ramalho Ribeiro, Carla Patinha, Artur M. S. Silva, Susana M. Cardoso, Rui Costa

**Affiliations:** 1 QOPNA and LAQV-REQUIMTE, Department of Chemistry, University of Aveiro, 3810-193 Aveiro, Portugal; 2 Polytechnic Institute of Coimbra, College of Agriculture, Research Centre for Natural Resources, Environment and Society (CERNAS), Bencanta, 3045-601 Coimbra, Portugal; 3 GEOBIOTEC, Department of Geoscience, University of Aveiro, Campus Universitário de Santiago, 3810-193 Aveiro, Portugal

**Keywords:** *Fucus sp.*, brown macroalgae, water extraction, bioactive, fucoidan, phlorotannin, kinetics

## Abstract

Brown macroalgae, particularly those from *Fucus* genus, are a rich and balanced source of bioactive nutrients and phytochemicals, such as dietary fibres (fucoidans, laminarins, and/or alginates), phlorotannins, and fucoxanthin, and some minerals, such as iodine, which have been demonstrated to possess numerous health-promoting properties. In fact, aqueous extracts of *Fucus vesiculosus* have been used as food supplements due to its rich content in bioactive compounds, though no study has been published on the optimization of this operation. Therefore, this study aimed to evaluate the impact of different extraction temperatures (25 °C, 50 °C, 75 °C, 100 °C, and 120 °C) and times (5 min, 1 h, 2 h, and 4 h) on the recovery of those bioactive compounds. The temperature was observed to positively influence the extraction of crude mass and of fucose polysaccharides only at 75 °C and above, and of iodine extraction at 50 °C and above. At these temperatures, time also showed to increase yields. Yields of crude extract, fucose, and iodine were successfully mathematically modelled with a power law, and its maximum yields were obtained at the highest temperature studied (120 °C) and longest extraction time (4 h). Iodine yield at these conditions provided extracts with relevant content to contribute to the recommended daily ingestion. Phlorotannins were significantly recovered at 120 °C though evidence of degradation was observed during time.

## 1. Introduction

The well-known prediction of global population of 9 billion people by 2050 requires an increased food production by more than 60% [1]. This puts pressure on natural resources sustainability and climate changes, leading to instabilities in primary production, food processing, and distribution. So, in a world of constant growth and change, a great deal of attention from the scientific community is being directed at marine environments, given that they comprise approximately half of total global biodiversity [2]. Overall, foods with marine derived ingredients can provide required amounts of nutrients and phytochemicals that are essential for the maintenance of healthy living [3]. In particular, seaweeds are considered a rich source of fibres and other carbohydrates, proteins, minerals, polyunsaturated fatty acids, vitamins, and phenolic compounds [4], and several scientific studies point to the fact that the intake of seaweeds may contribute to the prevention of various disorders and/or diseases, in particular, metabolic disorders, including obesity, diabetes, and hypertension [4,5,6,7]. 

All the referred facts are pushing the western culture to increase interest in the manufacturing and consumption of high-value products derived from macroalgae, with the main aim of taking advantage of their potential health effects. This interest is translated into a global functional food market $161.49 billion value by 2018 and forecasted to be more than $275.77 billion by 2025 [8], where opportunities are foreseen for applications of seaweeds, crude extracts, or its purified fractions.

The genus *Fucus* currently comprises 66 taxonomically accepted species, with *F. vesiculosus* being the most prominent. This species dominates the shallow macroalgae communities growing on high salinity waters from 0.5–4 m in depth and forms large belts that constitute the habitats for communities of epiphytic and epibenthic organisms [9]. *Fucus* species have been consumed as foods in coastal countries of Western Europe and Alaska [10]. Currently, there are many other uses for *F. vesiculosus* besides food ingredients, such as the production of soap, paper, glass, biofertilizers, animal feed, phytopharmaceuticals, among others [9].

Like other *Phaeophytae*, *F. vesiculosus* is fertile in distinct health-promoting compounds, such as fucoidans, fucoxanthin, phlorotannins, and essential minerals, particularly iodine [11,12]. Fucoidans are metabolites belonging to the fucans family and stand out as one of the bioactive compounds that are exclusive to brown algae [6]. Those obtained from *F. vesiculosus* were shown to be composed of 44.1% fucose, 26.3% sulfate, and 31.1% ash, with the main component unit of 1,3-α-fucose, while sulfate groups are mostly linked at position C-4 of this sugar [13]. These polysaccharides are claimed to exert antioxidant [14,15,16], anti-obesity [17,18,19], and anti-diabetic [20,21,22] activities, but others, such as anti-aging, antimicrobial, antitumor, anticoagulant, anti- inflammatory, and contraceptive effects are also reported [23]. 

As a part of the tannins group, phlorotannins present a polymeric structure and possess a high number of hydroxyl groups, being therefore highly soluble in water. These represent the major phenolic compounds of brown algae and, particularly in *F. vesiculosus*, may represent up to 12% of their dry weight [9]. They are involved in the biosynthesis of some components of the cell wall and work as defensive mediators against natural enemies, working as herbivore deterrents, digestive inhibitors, and as antibacterial and antifouling agents [24]. Furthermore, they have been shown to exert numerous biological activities, including antioxidant, antibacterial, and antidiabetic activities [7,24,25].

Due to their specific structural and physiological features, brown macroalgae, like *F. vesiculosus*, are recognized for their superior ability to accumulate minerals. In this referred study, *F. vesiculosus* is claimed to possess relatively high amounts of minerals like calcium, magnesium, phosphorus, potassium, sodium iron, and particularly iodine [12]. Besides, brown macroalgae are characterized by low Na/K ratios, which is an important aspect for good maintenance of cardiovascular health [26]. Also, iodine is not widely available in foods [27] and its consumption is essential for the synthesis of thyroid hormones, and is responsible for the regulation of the metabolism and the development and maturation of the organs [28]. 

Considering the high concentration of bioactive compounds present in *Phaeophyta*, and particularly in *F. vesiculosus*, their extraction has been tested with several techniques to obtain high yields of extraction, as well as high quality of extracts (higher concentration of bioactive compounds). Polysaccharides from brown seaweeds have been mainly extracted with multi-step and chemical extractions using acidic or alkaline media, or calcium chloride [29]. Moreover, many reports have used techniques that combine the use of organic solvents and water (acetone 70%, methanol and ethanol 80%, among others) with long extraction times, high temperature, and the use of enzymes to digest other cellular components (pepsin, pancreatin, for example) [9]. However, it has been noted that although the use of caustic chemicals increases the extraction yields, it may change the structure of seaweeds’ native molecules, which in some case can alter its function [30]. New methods, such as supercritical fluid extraction, subcritical water extraction, ultrasound-assisted extraction, microwave-assisted extraction, and autohydrolysis have also been studied with high yields being reported [31,32], however, these methods are not suitable for an industrial scale yet. 

Water extraction is the simplest and most feasible extraction method to implement at an industrial scale, but still, much is to be known on designing the processing with optimum conditions. In particular, extraction of bioactive compounds from *F. vesiculosus* was only studied to a limited extent, in a short range of temperatures and operation times [33,34]. However, industrial applications will take advantage from a wider knowledge of aqueous extraction kinetics for optimization of bioactive compounds extraction and process sustainability, where low cost, secondary streams minimization, and batch variability must be tackled. For that reason, the main aim of this work was to study the kinetics of water extraction of bioactive compounds from *F. vesiculosus* in a wide range of temperatures and operation time.

## 2. Results and Discussion 

### 2.1. Mass Yield of the Aqueous Extracts 

**Figure 1:**
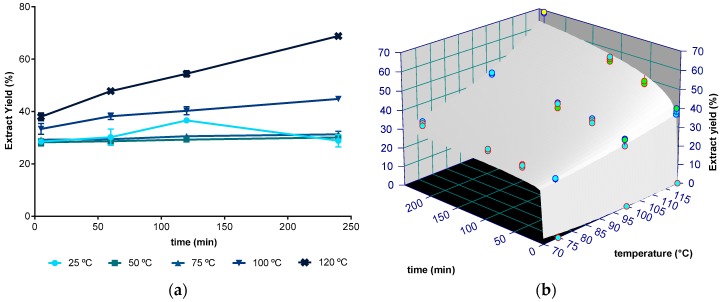
(**a**) Aqueous crude extract yield (g of dried extract/100 g of dried seaweed) at 25, 50, 75, 100, and 120 °C between 5 minutes and 4 hours. (**b**) Fitting plot of power law with Arrhenius dependence to the crude extract at 75, 100, and 120 °C during 5 min to 4 h (240 min) (blue, green, and yellow, identify, respectively, 1, 2, and 3 standard deviations).

The mass yield of aqueous extracts obtained for distinct extraction times (5 min, 1 h, 2 h, and 4 h) and for temperatures between 25 and 120 °C is shown in Figure 1a. Values ranged between 26 g of dried extract per 100 g of dried seaweed weight (DW) at 25 °C, to 69 g/100 g DW at 120 °C after 4 h of extraction. Note that, as reported before, the kinetics of crude extracts typically shows an initial quick step, known as washing stage, followed by a slow step, named diffusion stage (in this work observed up to 5 min and from 5 min onwards, respectively) [35]. The washing step is convection dependent [36] and may take just seconds, enough time to complete immersion, but the minimum time tested in this work was 5 min.

In general, the yield of extraction was not statistically different (*p* > 0.05, one factor ANOVA) for any temperature and time defined in the 25 to 75 °C range, suggesting that at these temperatures, only the washing phase is visible for extractions up to 4 h. The diffusion phase is responsible for significant extraction at 100 and 120 °C, whose yields increased with increasing extraction time (*p* < 0.05). Although the results at 2 h/25 °C were statistically different from other extracts obtained at the same temperature, this was considered an experimental error, as the expected kinetics is an increasing yield for longer times for all temperatures, as observed for the other temperatures (Figure 1a).

The kinetics of the crude extract yield over time were modelled with a power law model (see 3.4). This model has two parameters: *k*, which is related to the amount of extract in the washing phase and kinetic information, and *n*, which is related to the rate change with time. Figure 1b presents the fitting of the experimental data to the power law model applied to temperatures that yielded significant differences (i.e., between 75 and 120 °C) and the resultant parameters are presented in Table 1. The fitting quality evaluated by the coefficient of determination (R^2^) allows the conclusion that the power model law provides reasonable adjustments and the possibility of making good predictions of the crude extract yield from milled seaweed. However, the model presented lack of fit (*p* < 0.05), indicating that the prediction is biased. This model also presents overfitting with *n*_0_ having a high standard error, meaning that changes in this parameter will not significantly affect the results predicted by the mathematical model used. The washing phase magnitude given by the parameter *k* is affected more by temperature than the time related parameter *n*, as given by the respective activation energies of 3.17 kJ/mol and 30.62 kJ/mol. 

Other mathematical models besides the power law could also be used in this work [35], such as the empirical Peleg model, which has been used to predict extraction [37], but one must note that parameters following the Arrhenius dependence with temperature would require the estimation of a total of six parameters, which, applied to this limited number of experimental data (four times per temperature), would result in excessive overfitting [38], leading to large standard errors in the parameter estimation (data not shown). On the other hand, using a single parameter model (such as C = k × t^1/2^), the loss of prediction accuracy is tremendous, with coefficients of determination getting closer to 0.9.

Extraction yields and rates depend on several factors, the subtract (solid phase) and the solvent being two of the most important [35]. The solid phase is known to be affected by the disruption of cell walls and lysis of cytoplasm, diffusion coefficient from inner cells to the extraction medium, and desorption from cell walls. The influence of the solvent phase depends on the polarity of the extractables and solvent, the solubility of extractables in the solvent, and the solvent viscosity and surface tension. All these phenomena are accelerated (time dependent) and magnified by temperature. In general, extractions yields are incremented with higher solubility, which is promoted at higher temperatures. Additionally, desorption, mass transfer, and reaction rate dependent phenomena show larger effects with increasing extraction times [39]. Based on this knowledge, it is expected that water-soluble components are recovered at higher yields for longer times of extraction and at higher temperatures. For total mass extraction, the results obtained in this work confirm that prediction, but only at temperatures higher than 75 °C, and thus the influence of time was only significant at higher temperatures. When considering the mean chemical composition of *F. vesiculosus* (i.e., 4–59% DW of fibers (from which approximately 12.4–91.5% DW are soluble), 1–11% DW of proteins and 23–36% DW of minerals [11]), it is feasible to hypothesize that aqueous extracts might contain several of these components in considerable amounts, and that the crude extract mass will depend mainly on the extraction of those major compounds. 

### 2.2. Fucose 

**Figure 2:**
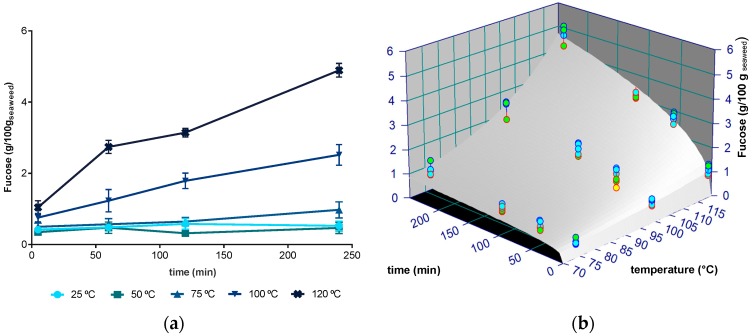
(**a**) Amount of fucose recovered from *F. vesiculosus* (g/100 g of dried seaweed) at 25, 50, 75, 100, and 120 °C between 5 minutes and 4 hours); (**b**) Fitting plot of power law with Arrhenius dependence to the fucose extract at 75, 100, and 120 °C during 5 min to 4 h (240 min) (blue, green, and yellow, identify, respectively, 1, 2, and 3 standard deviations).

Fucose was used in this work as an indicator of fucose-containing sulphated polysaccharides, particularly fucoidans, which are known to be abundant in *Fucus sp.* According to our results (Figure 2a), the amounts of fucose recovered from *F. vesiculosus* varied between 0.0911 g/100 g DW to 5.075 g/100 g DW, with maximum values being obtained at 120 °C during 4 h. Globally, the extraction of fucoidans followed a close behaviour to that noticed for the yield of extraction, i.e., no significant changes were registered between 25 and 75 °C for any extraction time, while a significant yield increment was observed for 100 and 120 °C and for longer times at these temperatures. This fact was reflected by a correlation coefficient close to 1 (r = 0.964, *p* < 0.001) between these two parameters and suggests that fucoidans are major components of the resulting extracts. 

As for the total extract yield, the power law model was adjusted to the experimental data and applied at temperatures that showed significant differences (i.e., between 75 and 120 °C). The fitting to experimental data is presented in Figure 2b and the parameters are summarized in Table 1. The power law model enabled good quality predictions of fucose recovery from *F. vesiculosus*, for temperatures of 75 to 120 °C and at all times (R^2^ = 0.9532). From 25 to 75 °C, the average extraction yield was 0.522 g/100 g DW (±0.209 g/100 g) for any extraction time. As for the extract yield fitting, the model presented lack of fit (*p* < 0.05) and overfitting with *n*_0_ and *k*_0_ having large standard errors. For fucose, the activation energies of *k* and *n* were similar and low (14.12 and 14.78 kJ/mol, respectively), indicating that both the magnitude of the washing phase and the kinetic rate suffer similar and sharp influence of temperature.

The amount of recovered fucose increased with the temperature of extraction, being more than five times higher at 120 °C than at 25 °C. These results support those previously obtained by Jiao et al. [40], who reported an increment of about 50% on the recovery of fucoidans from *F. vesiculosus* with water at 80 °C comparing to a room temperature extraction for 3 h. On the other hand, when working with *F. vesiculosos*, Rupérez et al. [41] observed that the amount of fucose extracted was lowered by about 3.3% at a higher temperature (60 °C), compared to extracts obtained at 22 °C. Rodriguez-Jasso et al. [32], also working with the same seaweed, observed that the fucose content in aqueous extracts decreased by 28.95% when the extraction temperature was raised from 160 to 200 °C, while Ale et al. [29] reported that that longer extraction times resulted in different composition of fucoidans. Hence, overall, the reported results suggest that the use of high temperatures of extraction may result in the partial degradation of fucoidans, albeit a solid conclusion cannot be taken because authors always used acidic conditions (≥0.2 M HCl) [42]. Notably, some authors also registered differences in the relative composition of sugars in both extracts [41]. Nevertheless, Hahn et al. [42] concluded that mild aqueous extraction conditions are more suitable to obtain the native polysaccharide fucoidan, avoiding the loss of the sulphate and its structural alteration. Studying at several temperatures up to 90 °C, these authors found a maximal fucoidan yield of 7% DW achieved at 4 h with an acid concentration of 0.03 M HCl.

### 2.3. Iodine 

**Figure 3:**
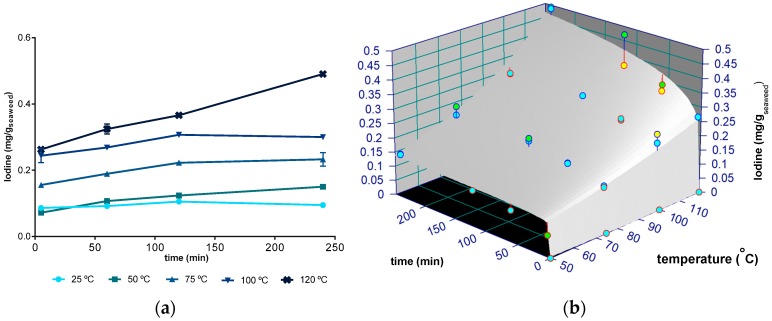
(**a**) Amount of iodine (mg/g of dried seaweed) extracted at 25, 50, 75, 100, and 120 °C and between 5 minutes and 4 hours; (**b**) Fitting plot of power law with Arrhenius dependence to the iodine extract at 50, 75, 100, and 120 °C during 5 min to 4 h (240 min) (blue, green, and yellow, identify, respectively, 1, 2, and 3 standard deviations).

The recovery of iodine in an aqueous extraction, for times ranging from 5 min to 4 h and temperatures between 25 and 120 °C, is shown in Figure 3. The values varied from 0.072 mg/g DW at 50 °C/5 min to 0.4903 mg/ g DW at 120 °C/4 h of extraction, which means that the use of the latter condition, about 0.3 g of algae extract, would be sufficient to provide the recommended daily amount of iodine for an adult to ingest (0.150 mg/day). Moreover, when comparing the amounts of iodine extracted at 120 °C/4 h with the values of iodine in *F. vesiculosus* [43], it is possible to suggest that the latter conditions allowed recovering almost all the iodine present in this algae. 

According to our study, the recovery of iodine did not change significantly (*p* > 0.05) between 25 and 50 °C (average of 0.104 ± 0.024 mg/g DW). In turn, for temperatures between 50 and 120 °C, the amount of iodine extracted from *F. vesiculosus* increased with the time studied (*p* < 0.05). Hence, opposing to the mass and fucoidans yields (for which the lowest temperature limit to observe changes was set at 75 °C), the herein gathered data indicate that the washing phase for iodine extraction is less relevant. 

Iodine can be presented in various forms, from inorganic (iodide) to organically-bound, mostly to amino acids [44]. Up to 99% can be extracted by water, in the form of iodide (61–93%), organic iodine (5–37%), and iodate (1.4–4.5%) [43]. It is possible that the washing phase may leach both forms simultaneously, but especially the inorganic form that, as a smaller particle than the iodine-organic molecule complex, will diffuse faster. Moreover, as reported by Romarís-Hortas et al. [43] for nori seaweeds, organic iodine seems to be further extracted by alkaline solvent. Instead, the water used as a solvent in our study had an acidic pH (4.9).

The power law model was also used to fit the experimental results of iodine extraction and presented a reasonable fitting (R^2^ = 0.9655, Table 1), enabling predictions of the recovery of iodine from milled seaweed for the temperatures 50 to 120 °C. As for the crude extract yield and fucose fitting, the model presented lack of fit (*p* < 0.05) and overfitting with *n*_0_ estimated with a large standard error. The influence of temperature on iodine extraction, given by the activation energies (Table 1), is similar to its influence on the crude extract yield, i.e., a higher influence on the washing phase extraction magnitude than on the influence of time. Since no study was found on the extraction kinetics of iodine from seaweeds, no comparisons on the extraction kinetic can be made.

### 2.4. Phlorotannins

**Figure 4:**
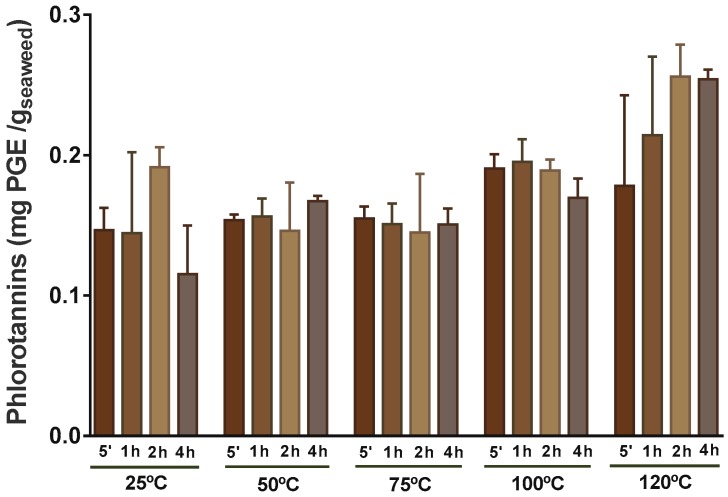
Levels of phlorotannins for extraction at 25, 50, 75, 100, and 120 °C between 5 min and 4 h expressed as mg of phloroglucinol equivalents (PGE)/g of dried seaweed.

Phenolic compounds are usually more soluble in apolar solvents or solvents less polar then water, therefore the most common extractants used for these compounds are methanol, ethanol, and acetone, or aqueous mixtures of these [24,45]. Such mixtures are used to extract those soluble in water. However, because of safety concerns regarding the use of some organic solvent extracts in foods, for this work, water was selected for extraction of the phlorotannins. Furthermore, once there was no available information regarding the kinetics of extraction of phlorotannins in *F. vesiculosus*, the extraction of these phenolic compounds was studied, as can be observed in Figure 4, for five different temperatures and times. 

The amount of phlorotannins extracted from *F. vesiculosus* ranged between 0.115 mg of phloroglucinol equivalents (PGE)/g DW at 25 °C and approximately two times more, 0.226 mg PGE/ g DW at 120 °C (Figure 4), allowing the conclusion that the highest temperature was the most promising to recover these phenolic compounds from *F. vesiculosus*. Yet, one must highlight that the resulting extracts at 120 °C, particularly that obtained at 4 h, tended to be poorer in phlorotannins (Figure S1), suggesting that the rate of extraction of these compounds under such conditions was surpassed by others (e.g., polysaccharides). In fact, the most rich extract in terms of phlorotannins was herein obtained at 50 °C and 100 °C (extracts obtained at 4 h and 5 min, respectively) corresponding to 0.056 mg PGE/mL (equivalent to 0.56 g PGE/100 g extract), a value that corresponds to about half that reported by Agregán et al. [33], when extracting *F. vesiculosus* with water at 40 °C for 5 min to obtain a correct hydration and 10 min in an ultrasonic homogenizer. Naturally, when comparing our results with those obtained using organic solvents, the difference in the level of phlorotannins extracted is even higher. Catarino et al. [7], using different ratios of acetone:water, found that the use of 10% of acetone at room temperature for 24 h allowed the recovery of 0.77 ± 0.05 mg PGE/g DW from *F. vesiculosus*, a value that corresponds to approximately 3.5 times higher than the maximum obtained in this work (0.256 mg PGE/ g DW). Nevertheless, it is notable that, when extracting *F. vesiculosus* with water at room temperature for 1 h, Koivikko et al. [46] obtained a high yield of phlorotannins in the resulting extract (more than 4% DW of the extract), only exceeded by the extraction with a mixture of 70:30 acetone:water [25]. 

*F. vesiculosus* has been reported to have an array of phlorotannins of less than 1.2 kDa [47], thus corresponding to the lower range of sizes of these compounds, which are known to reach more than 100 kDa [48]. So, considering the molecular weight of phlorotannins described to occur in *F. vesiculosus*, it is probable that they can readily be transferred to the water extract, which may explain the lack of variation during time with any temperature tested (Figure 4).

Using ANOVA, it was found that the phlorotannin extraction was significantly different (*p* < 0.05) between the range of lower temperatures (25 °C, 50 °C, and 75 °C) in comparison to 120 °C (Figure 4), though experimental data presented great variability. Furthermore, no significant influence of extraction time was observed at any temperature tested. For that reason, no mathematical model was used to predict the extraction along time. 

Other published works on phlorotannins’ water extraction can be found in literature, although these only used a low range of temperatures, hampering a valid comparison with our results. Besides, the influence of temperature was also studied with mixtures of solvents at lower temperatures. Li et al. [49] observed that the extraction efficiency of phlorotannins from *Sargassum fusiforme* in ethanol increased significantly from 15 to 25 °C, but then decreased significantly from 25 to 55 °C. Catarino et al. [7] also found a decrease above 25 °C, extracting from *F. vesiculosus* with mixtures of water and acetone. The decreases were attributed to degradation, such as the oxidation. Boi and Cuong [50], working with *Sargassum serratum* (brown seaweed), found that the degradation of phlorotannins at extracting temperatures higher than 50 °C was due simultaneously to coagulation and to a change in the structure of the cells. Once the phlorotannins exist in a free or *quasi*-free state, they will be affected by oxygen, leading to structural changes [51]. These mechanisms suggest that the results obtained in this work might be a net balance between the higher extraction of phlorotannins at higher temperatures and longer times, and a degradation of these compounds due to exposure to the same high temperatures simultaneously with exposure to oxygen, resulting in a high variability of extraction yields at the same time–temperature.

### 2.5. 2,2′-Azino-bis(3-ethylbenzothiazoline-6-Sulphonic Acid (ABTS^•+^) Assay

**Figure 5:**
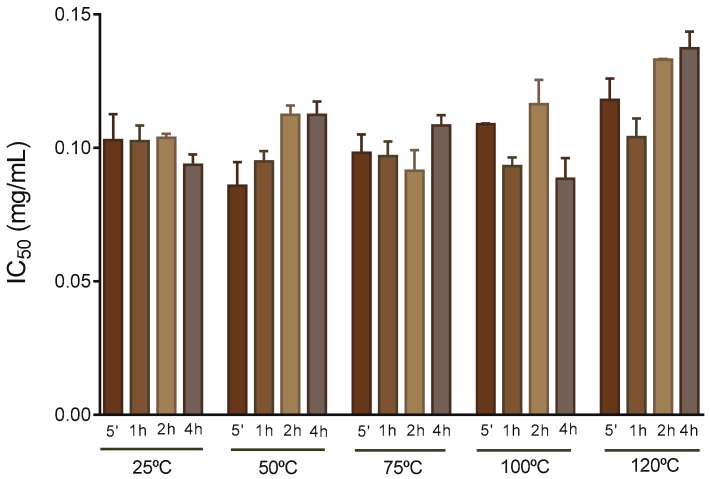
ABTS^•+^ scavenging ability of aqueous extract obtained at 25, 50, 75, 100, and 120 °C between 5 min and 4 h, expressed in IC_50_ (mg/ mL of extract).

In general, polar extracts obtained from *F. vesiculosus* possess high antioxidant activity [25]. In this regard, Agregán et al. observed that a *F. vesiculosus* water extract produced at 40 °C presented a higher antioxidant activity than the equivalent extracts obtained from *Ascophyllum nodosum* and of *Bifurcaria bifurcata,* as measured by ABTS^•+^, 2,2-diphenyl-1-picrylhydrazyl (DPPH^•^), and fluorescence recovery after photobleaching tests [33]. In this work, the antioxidant activity of the extracts was evaluated by means of ABTS^•+^ scavenging ability, as represented in Figure 5.

The extracts showed no significant differences (*p* > 0.05) regarding the ability to scavenge the ABTS radical (IC_50_ values of 0.088–0.134 mg/mL), though the average value at 120 °C was slightly higher (slightly lower antioxidant activity). This last tendency is consistent with the lowering of pholorotannins in the extract (Figure S1), suggesting that pholorotannins are key players in the antioxidant properties of the extracts. In fact, the direct correlation between the concentration of phlorotannins and antioxidant activity has been previously reported by distinct authors, including Liu and Gu, when working with several extracts from *F. vesiculosus* [45]. Nevertheless, one must note that several other compounds present in *F. vesiculosus* have also been attributed to have antioxidant activity, such as fucoidans and carotenoids [11], which can explain the almost reduced variation in the ABTS^•+^ scavenging ability for the time and temperatures tested once, at higher temperatures, higher amounts of fucoidans were extracted that could have a positive influence in the IC_50_ of the samples. 

When testing extraction from the seaweeds *Sargassum polycystum, Eucheuma denticulatum*, and *Kappaphycus alvarezii,* with a solvent mixture of water and alcohol, Fu et al. [52] obtained higher antioxidant activity of extracts by DPPH^•^ after extraction at higher temperatures at the range 25–65 °C but a decrease at 75 °C, a fact that the authors associated with degradation of phlorotannins at higher temperatures. Fu et al. also studied the influence of time (between 1 and 5 h), where an increase of ABTS^•+^ scavenging capacity was apparently observed for the seaweeds studied, but no statistical analysis was presented. Additionally, hot and cold-water extracts were found to have different bioactivity [34], with cold water extracts showing significant anti-inflammatory activity, an effect not observed in the hot water extract. This may well be the explanation for the results in this work; like for the pholorotannins extraction, the effect of a higher extraction of antioxidants at higher temperatures and longer times may be counterbalanced by its degradation at the same conditions, resulting in no significant differences along time and temperatures. 

## 3. Materials and Methods

### 3.1. Sample Collection 

The seaweeds from *F. vesiculosus* were cultivated in a land-based integrated multitrophic aquaculture (IMTA) system at ALGAplus Lda, a company based in Aveiro district, Portugal, specialized in seaweed cultivation and their commercialization into the food, cosmetics, and feed markets. After collection, the macroalgae were washed with sterilized seawater, followed by centrifugation to remove excess water. The seaweed was then dried at 20 °C and milled with Retsch SK10 to particles with less than 250 µm diameter. 

### 3.2. Extraction Experiments

Single extractions were done in triplicate at temperatures of 25, 50, 75, 100, and 120 °C in a non-stirred water bath PrecisionTerm Selecta (Barcelona) during 5 min, 1 h, 2 h, and 4 h using 7 g of seaweed powder and 140 mL of distilled water (pH = 6.6 at 25 °C) in 250 mL Duran flasks. The pH after extraction of all extracts were with the range 5.55–5.60. Duran flasks were closed during the extraction to avoid water vapour losses. Extraction at 120 °C was done in a retort (Raypa AES-75, Barcelona, Spain) with the same holding times, with additional an 30 min to heat the sample to 120 °C and 30 min to lower the temperature to 100 °C, after which it was immediately cooled down in a water bath. The extraction time of 4 h at 120 °C was thus not a continuous time at this temperature but included two additional temperature ramps for heating and cooling. Additionally, at 120 °C, the total pressure is twice the pressure at the other temperatures. However, according to the available literature no specific effect of pressure is expected to affect extraction of soluble materials at these pressure levels [53].

The solubilized material was separated from the sediments in a centrifuge at 20 °C, 6780 rpm for 10 min, followed by freezing and free-drying (UNICRO MC-4L-60 °C, Martinsried, Germany). The resulting dried extracts were weighted and kept frozen up until chemical analyses.

### 3.3. Chemical Analysis

#### 3.3.1. Fucose

Fucose (l-fucose) was determined using a commercial kit (l-fucose, Megabyte, Bray, Ireland). Briefly, 10 mg of extract was diluted in 1 mL of distilled water and if there was no complete dissolution, samples were vortexed and filtered (0.45 mm polytetrafluoroethylene (PTFE) syringe filters). Subsequently, 200 µL of water, 10 µL of fucose extract, 40 µL of fucose kit buffer, and 10 µL of nicotinamide adenine dinucleotide phosphate (NADP^+^) solution (supplied by the kit) were placed on a 96 polystyrene (PS) flat bottom well plate. After 4 min of incubation at room temperature, 5 µL of l-fucose dehydrogenase suspension (supplied by the kit) was added and the mixture was incubated at 37 °C for 10 min. Finally, the absorbance was measured at 340 nm using the Multiscan plate reader. Fucose was quantified using the standard supplied by the kit. The fucose content was expressed as g/100 g of dried seaweed [54].

#### 3.3.2. Assisted Alkaline Digestion 

A microwave assisted extraction (MAE) procedure was carried out by weighing 0.1 g of powdered seaweed into microwave Teflon vessels, and adding 5 mL of ultrapure water and 5 mL of tetramethylammonium hydroxide (TMAH). Each seaweed sample was microwave alkaline digested three times, and one reagent blank was also prepared for each simple set. Vessels were then capped and subjected to microwave irradiation using two steps. In the first step, a ramp time of 10 min was used to increase the temperature from 20 to 200 °C. A second step at 200 °C was applied for 5 min. After cooling down, sample extracts were centrifuged for 10 min at 3000 rpm, transferring the supernatant to 50 mL volumetric flasks. The solid residue was rinsed with a small volume of water, and after centrifugation the water rinses were combined with the above-mentioned supernatant. Finally, extracts were filtered through 0.45 µm cellulose acetate syringe filters (Millipore, Burlington, MA, United States) before ICP-MS measurements [55].

#### 3.3.3. Iodine

A 7700 inductively coupled plasma mass spectrometer (ICP-MS) from Agilent Technologies, equipped with nickel sampler and skimmer cones and a collision/reaction cell, was used for iodine determination. Rh was used as the internal standard. TMAH extracts were conveniently diluted before ICP-MS measurement and the dilution was varied from 1:5 to 1:20. Isotope of ^127^I was analyzed, with 3.0 mL/min of He as the reaction gas to avoid interferences on this mass. The limit of detection (LOD) and the limit of quantification (LOQ) of the ICP-MS measurements were 0.5 and 1.3 μg/L, respectively, for I. The accuracy of our method was validated by Seronorm TM trace elements Blood L2 standard reference material. The CRM determination value for I was 86.6 ± 5.8 ng/g, while the reference value on the certificate was 107 ± 22 ng/g.

#### 3.3.4. Determination of Total Phlorotannin Content 

Quantification of total phlorotannins was carried out according to the 2,4-dimethoxybenzaldehyde (DMBA) colorimetric method previously described [7]. Briefly, equal volumes of the stock solutions of DMBA (2%, *m*/*v*) and HCl (6%, *v*/*v*), both prepared in glacial acetic acid, were mixed prior to use (work solution). Afterwards, 250 μL of this solution was added to 50 μL of each extract in a 96-wells plate and the reaction was incubated in the dark, at room temperature. After 60 min, the absorbance was read at 515 nm in an automated plate reader (Synergy|HTX from BioTek, Winooski, VT, USA) and the phlorotannin content was determined by using a regression equation of the phloroglucinol linear calibration curve (0.06–0.1 mg/mL). The results were expressed as mg phloroglucinol equivalents/g dry seaweed (mg PGE/g DS).

#### 3.3.5. ABTS^•+^ Discoloration Assay

This method was performed according to the procedure of Catarino et al. [9] with some modifications, as described elsewhere. The ABTS^•+^ solution was prepared by reacting the stock solution of ABTS (7 mM) with potassium persulfate (2.45 mM) in a ratio of 1:1. The solution was stood in the dark at room temperature for 12–16 h. Before usage, the stock solution was diluted with ethanol to get an absorbance of 0.70 ± 0.020 at 734 nm. Several concentrations of sample extracts/standard were dissolved in 250 µL of diluted ABTS^•+^ solution. After 20 min of incubation, the absorption at 734 nm was measured using an ELX800 microplate reader. The percentage of inhibition was calculated using the equation:

ABTS^•+^ scavenging activity (%) = (Abs_control_ − Abs_sample_)/Abs_control_ × 100

where Abs_control_ is the absorbance of ABTS radical, the control without extract addition, and Abs_sample_ is the absorbance of ABTS radical with extract. The results were expressed as IC_50_ (concentration of the extract able to inhibit the 50% of the ABTS^•+^) of each extract. Ascorbic acid was used as a positive control for comparison. 

### 3.4. Statistical Analysis and Mathematical Modeling

Data were subjected to a two-factor and a one-factor analysis of variance. For data with non-homogeneity of variances, the Kruskal–Wallis non-parametric test was performed. Following ANOVA, means were compared by the Tukey’s multiple comparison test. Statistical significance was tested at 0.05 probability level. All statistical tests were performed using the SPSS software V 25.0.0 (IBM, Armonk, NY, USA). 

The power law model was applied to temperatures that yield significant differences for crude extract, fucose recovery, and iodine extraction, as follows:

$$C=kt^{n}$$

where *C* is the yield of the component being extracted (%, g/100 g seaweed), *t* is the time (h), and *k* (% t^−n^) and *n* the kinetic parameters. This model is the result of two transport mechanisms, Fickian diffusion and Case-II transport [56], with both parameters being related to transport parameters [57]. Additionally, k includes equilibrium information, hence the units containing %.

Arrhenius dependence was applied to the kinetic parameters *k* and *n*:

$$k=k_{0}e^{-\frac{Ea}{RT}}$$

$$n=n_{0}e^{-\frac{Ea}{RT}}$$

*Ea* is the activation energy (J/mol), *k*_0_, *n*_0_ are constants, and *R* is the ideal gas constant (8.314 J/mol·K).

Table Curve 3D v4.0 (Systat Software Inc, San Jose, CA, USA ) was used for the fitting of the mathematical model applied to crude extract yield, fucose, and iodine kinetics, for parameter estimation, its standard error, coefficient of determination (R^2^), and lack of fit test. Fitting criteria used was the minimization of sum of square of residuals, with direct matrix solution methods to estimate coefficients and the lower-upper decomposition to estimate errors and confidence intervals. All the parameters were determined with simultaneous regression with independent variables time and temperature, and yield as the dependent variable.

## 4. Conclusions

**Figure 6:**
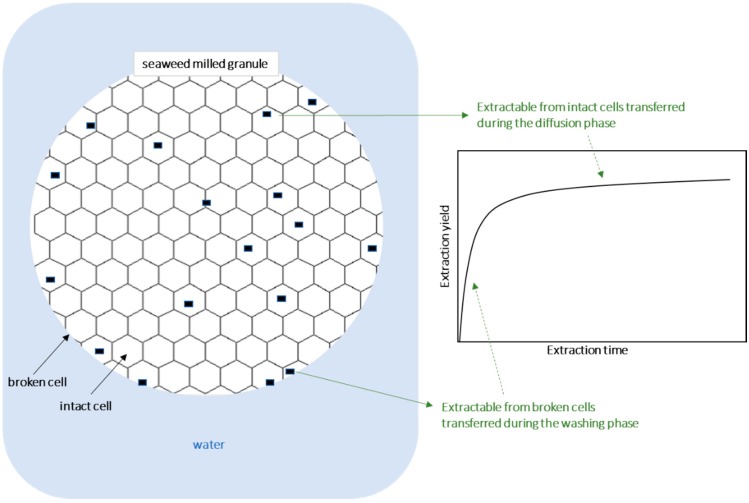
Illustration of the extraction phases.

It was expected that water soluble bioactive compounds from *F. vesiculosus*, namely fucoidans, iodine, and phlorotannins/antoxidant compounds, would be more extracted at higher temperatures and longer times, in two phases, corresponding to a washing phase and diffusion type phase (Figure 6). This work enabled us to know the magnitude of those effects and to model it for future optimization of the extraction at a lab or industrial scale. At the lower temperatures (depending on the extractable) and during all times studied, the extracted compounds were only recovered during the washing phase, since no influence of time on the yields was observed. Only for the highest temperatures the diffusion phase was relevant to contribute to the extracted material, leading to an increase of yields for a longer extraction time. Power law was observed to deliver predictions with good accuracy of extraction of the crude extract, fucose, and iodine. Extraction of these compounds was higher at 120 °C than at the other temperatures. For extraction times up to 4 h, only temperatures of 50 °C or higher for iodine, or 75 °C or higher for crude extract and fucose were observed to present significant higher extraction during longer times. However, the extracts showed no significant differences regarding their antioxidant capacity, as assessed by the ability to scavenge the ABTS radical, a fact that is probably associated with their steady richness in phlorotannins. Phlorotannins extraction have higher extraction yields at higher temperatures and longer times, however, it appears that those compounds are degraded when exposed to the same high temperatures simultaneously with exposure to oxygen, resulting in a high variability of extraction yields at the same time–temperature. 

These results open the possibilities of implementing green extraction to bioactive compounds of *Fucus vesiculosus* at an industrial level. Water extraction of *F. vesiculosus* complies with several principles of green extraction [58]: (i) it provides a new renewable source of bioactive compounds, (ii) uses water instead of organic solvents, (iii) resulting in a non-contaminated product, (iv) if done at low temperatures prevents energy spending, (v) provides a single operation when compared to multiple extractions, and (vi) the extracted solid matter is suitable for the manufacturing of food products.

## Figures and Tables

**Table 1:** Parameters and their standard deviations of power law and Arrhenius dependence applied to iodine, fucose, and crude extract yield kinetics.

	T Range (°C)	*k*_0_ (g/100 g Seaweed)		*Ea_k_* (kJ/mol)		*n*_0_		*Ea_n_* (kJ/mol)		R^2^
Extract yield	75–120	76.05	±36.70	3.17	±1.47	1668	±2186	30.62	±4.22	0.9818
Fucose	75–120	36.1	±66.8	14.1	±5.9	37.9	±47.7	14.8	±4.1	0.9532
Iodine	50–120	3.19 × 10^−3^	±2.48 × 10^−3^	9.2	±2.3	35.7	±62.5	17.7	±5.6	0.9655

## Supplementary Materials

The following are available online at https://www.mdpi.com/1420-3049/24/18/3408/s1, Figure S1: Phlorotannins content extracted at 25, 50, 75, 100, and 120 °C between 5 min and 4 h are expressed as mg equivalents of phloroglucinol (PGE)/100 g of dried extract.
