# Supplementary figures and images for: Water Extraction Kinetics of Bioactive Compounds of Fucus vesiculosus
Source: Molecules. 2019 Sep 19;24(18):3408. doi: 10.3390/molecules24183408 (PMC6766934; doi:10.3390/molecules24183408)

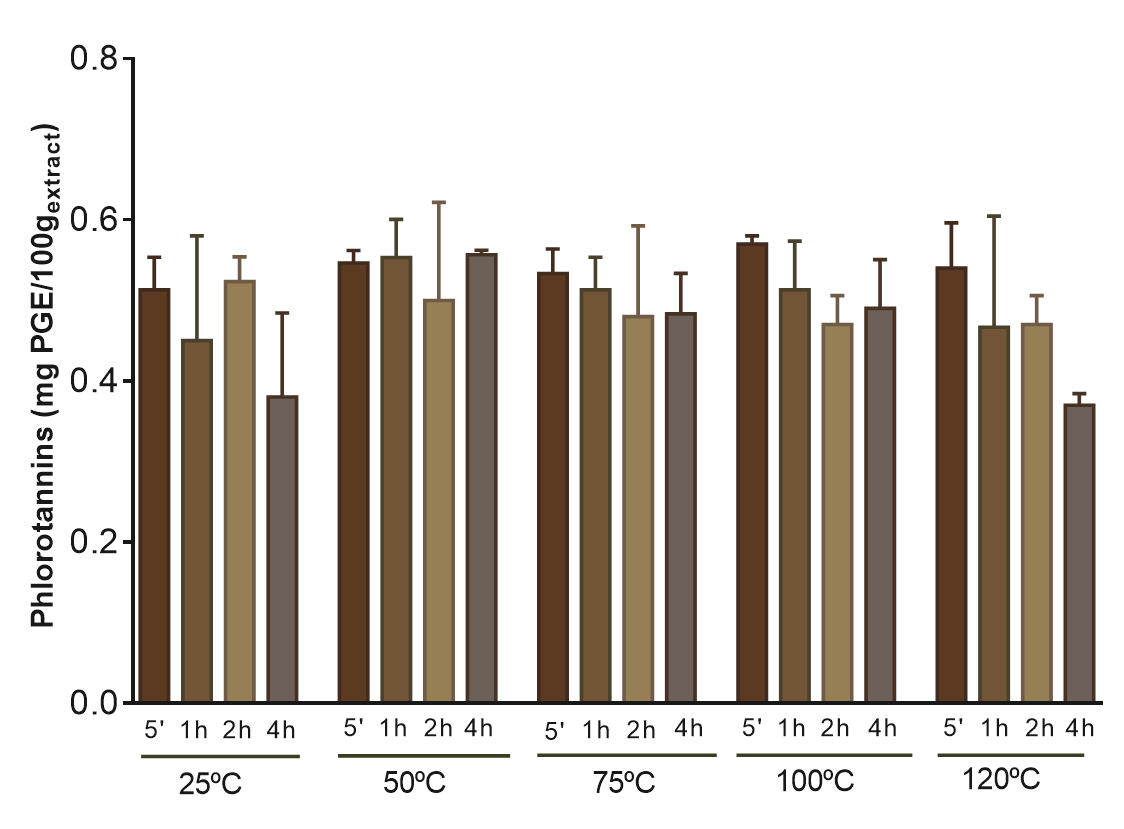

Supplement: Supplementary file 1 [file molecules-24-03408-s001.zip › molecules-575653-SI.png]
